# Lardaceous Scirrhoma of the Lung, Involving the First Rib, Clavicle, &c.

**Published:** 1845-01

**Authors:** 


*Lardaceous Scirrhoma of the Lung, involving the First Rib, Clavicle, &c.*—A warper, 41 years of age, came under Dr. Tinniswood's care, having had cough and dyspnœa for twelve months, together with mucous expectoration, occasionally tinged with blood, and dull respiration on the right side, puerile and sonorous on the left. There was a large hard tumour on the right side of the neck, apparently rising from the first rib and clavicle. This had existed six months. This tumour continued to enlarge: the face and arms became anasarcous, and the veins of the breast tortuous and enlarged. About three weeks before death, the clavicle fractured. On examination of the body, the clavicle was found involved in a lobulated tumour, which extended up the neck, and encroached upon, and somewhat displaced, the vessels of the neck. The subclavian and innominata arteries passed through it; they were somewhat thickened and enlarged, whilst their accompanying veins were almost obliterated, especially the internal jugular, which was a mere thread. The tumour occupied all the left side of the superior thoracic opening, passing up from the lung, in the substance of which it seemed to have originated; the upper lobe being entirely occupied by it, whilst the middle was gradually assuming the same character, being condensed and completely solidified. The neighbouring muscles were also being involved in the diseased growth, and the cells of the first rib and manubrium of the sternum were filled with scirrhous matter. The clavicle itself had been gradually absorbed in the tumour, till a mere spicula remained, which had at last given way. The bronchi of the left lung were considerably dilated.—*Ibid.*

